# Deep brain stimulation of the medial geniculate body for refractory tinnitus: A feasibility study

**DOI:** 10.1016/j.neurot.2025.e00783

**Published:** 2025-11-18

**Authors:** Shabnam Babakry, Jana V.P. Devos, Catharine A. Hellingman, Linda Ackermans, Jasper V. Smit, Michelle Moerel, Carsten Leue, Annelien A. Duits, Yasin Temel, Marcus L.F. Janssen, the DBS Tinnitus Study Group, Pia Brinkmann, Erwin L.J. George, Sonja A. Kotz, Mark J. Roberts, Michael Schwartze

**Affiliations:** j Faculty of Psychology and Neuroscience, Maastricht University, Maastricht, the Netherlands; k Department of Otorhinolaryngology and Head and Neck Surgery, Maastricht University Medical Centre, Maastricht, the Netherlands; a Department of Neurosurgery, Maastricht University Medical Centre, Maastricht, the Netherlands; b Mental Health and Neuroscience Research Institute (MHeNS), Maastricht University, Maastricht, the Netherlands; c Department of Otorhinolaryngology and Head and Neck Surgery, Maastricht University Medical Centre, Maastricht, the Netherlands; d Department of Psychiatry and Psychology, Maastricht University Medical Centre, Maastricht, the Netherlands; e Department of Ear, Nose, and Throat/Head and Neck Surgery, Zuyderland Medical Centre, Heerlen, the Netherlands; f Department of Cognitive Neuroscience, Faculty of Psychology and Neuroscience, Maastricht University, Maastricht, the Netherlands; g Maastricht Centre for Systems Biology, Faculty of Science and Engineering, Maastricht University, Maastricht, the Netherlands; h Department of Medical Psychology, Maastricht University Medical Centre, Maastricht and Radboud University Medical Centre, Nijmegen, the Netherlands; i Department of Clinical Neurophysiology, Maastricht University Medical Centre, Maastricht, the Netherlands

**Keywords:** Deep brain stimulation, Medial geniculate body, Tinnitus, Pilot study, Neurosurgery

## Abstract

Tinnitus disorder can have a significant negative impact on quality of life, especially when refractory to standard care. Deep brain stimulation (DBS) of the medial geniculate body (MGB) attenuates pathological neuronal activity in the central auditory pathway and is a potential treatment for severe tinnitus. The aim of this pilot study was to assess the safety and feasibility of bilateral MGB DBS in patients with refractory tinnitus disorder. This randomised double-blind 2 ​× ​2 cross-over study was conducted at Maastricht University Medical Centre, Maastricht, the Netherlands. The included patients had treatment refractory, severe, and chronic tinnitus without an anatomical substrate. Patients with bilateral MGB DBS were randomised to an ON-OFF or OFF-ON stimulation order for two cross-over phases. Primary outcomes consisted of safety and feasibility. Secondary outcomes on tinnitus severity, psychiatric and cognitive functioning and quality of life were assessed at screening, after both cross-over phases and at one-year follow-up. Four patients were included. No irreversible stimulation-induced side effects were observed. Surgical-related side effects were transient and resolved within two weeks. All patients experienced DBS as an acceptable treatment. Three of four patients showed improvement of tinnitus complaints based on the Tinnitus Functional Index. In the non-responder, electrodes had the largest distance from the centre of the MGB. To conclude, this study shows that bilateral MGB DBS is safe and feasible for patients with refractory tinnitus. Findings suggest the potential for clinically meaningful reduction in tinnitus burden through DBS. Effectiveness needs to be further evaluated in a follow-up study.

## Introduction

Tinnitus is the auditory perception of a phantom sound without an external source. While the exact pathophysiology is still unknown, auditory deafferentation is thought to play a key role. The common hypothesis is that cochlear or peripheral nerve damage leads to decreased auditory input, potentially inducing pathological neuronal hyperactivity of the central auditory pathway, which is then falsely perceived as sound [1]. The prevalence of tinnitus is estimated at 14 ​% globally, with 2 ​% experiencing a severe form [2]. Tinnitus disorder differs from symptom-related tinnitus based on emotional distress, cognitive dysfunction, and/or autonomic arousal, which can lead to behavioural changes and functional disability [3]. Associated symptoms, such as insomnia and concentration difficulties, and psychiatric comorbidities can significantly impair quality of life (QoL) [4,5] and impose a substantial economic burden with societal relevance [6]. Current standard treatments include hearing restoration and cognitive behavioural therapy [7]. However, even after standard care, a significant number of patients still suffer severely, indicating the need for additional effective therapies.

A potential treatment for this subset of patients is deep brain stimulation (DBS) [8]. DBS is an invasive neuromodulation technique where electrical stimulation is applied in subcortical brain areas [9]. DBS is a safe and well-established treatment for several brain disorders [10]. A significant number of patients with tinnitus are willing to undergo invasive treatments to reduce their tinnitus burden [11]. Previous studies suggested that the medial geniculate body (MGB) of the thalamus plays a significant role in tinnitus, as evidenced by altered neural activity, changes in neurotransmitter levels, and modifications in connectivity with both auditory cortical and limbic regions [12,13]. The pathological neuronal activity in the central auditory network in tinnitus may potentially be attenuated by MGB DBS. In an animal study, MGB DBS alleviated tinnitus-like behaviour [14]. Based on the key role of the MGB in the tinnitus network, and its accessibility via stereotactic surgery, we suggest the MGB as a potential DBS target for tinnitus treatment [15]. The aim of this pilot study was to primarily assess the safety and feasibility of bilateral MGB DBS in patients with severe tinnitus, refractory to standard care. As secondary outcome measures, tinnitus severity, hearing function, psychological and cognitive function, as well as QoL were assessed.

## Material and methods

### Study design

**Fig. 1:**
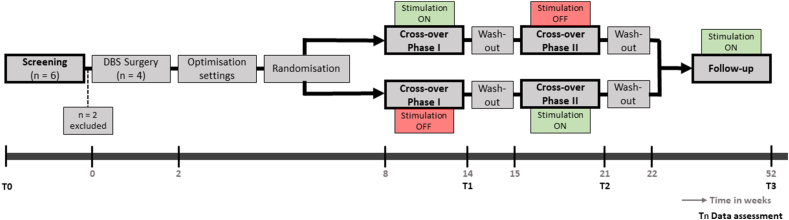
Overview of the study design. Randomisation was conducted based on a randomisation code offered by the independent CTCM. Patients and assessors were blinded to stimulation during two cross-over phases. ON refers to active DBS and OFF refers to no stimulation. Outcome measures were assessed at baseline (T0), after cross-over phase I (T1), cross-over phase II (T2) and 1-year follow-up (T3). The x-axis presents time in weeks. Outcome measures were tinnitus severity (TFI), hearing function (audiometry, ABR), cognitive functioning (Boston Naming Test, verbal and letter fluency, 15-Word Test, Trail Making Test and Stroop Color-Word Test) and psychological functioning including quality of life (SF-36), BDI-II, BAI, and HADS. In addition, a 3-T MRI was performed at screening and a CT-scan was performed during and after surgery. Adverse events and tolerability of the treatment were monitored throughout the whole study. Abbreviations: CTCM, Clinical Trial Centre Maastricht; DBS, Deep Brain Stimulation; TFI, Tinnitus Functional Index; ABR, Auditory Brainstem Response; SF-36, 36-Item Short Form Health Survey; BDI-II, Beck Depression Inventory II; BAI, Beck Anxiety Inventory; HADS, Hospital Anxiety and Depression Scale; MRI, Magnetic Resonance Imaging; CT, Computed Tomography.

This single-centre randomised double-blind 2 ​× ​2 cross-over study was conducted at Maastricht University Medical Centre (MUMC), Maastricht, the Netherlands. Ethics approval was obtained from the Institutional Review Board (NL67027.068.18, dated 06-08-2019). The trial was registered at ClinicalTrials.gov (NCT03976908, June 6, 2019). Privacy rights were observed and informed consent was obtained from all patients. The study consisted of different test phases (see Fig. 1 for further details). Outcome measures were assessed at baseline screening (T0), after both cross-over phases (T1 and T2), and a one-year follow-up (T3). Adverse events and tolerability of treatment were monitored throughout the study.

### Recruitment and selection

Patients were recruited at the outpatient clinic of the Otolaryngoly and Audiology Department (MUMC). All enrolled patients were diagnosed with treatment-refractory tinnitus, defined as non-responders to available care, following the Dutch Guidelines of Tinnitus [7]. After written informed consent was obtained, patients were screened to assess in- and exclusion criteria (see Table 1). Screening consisted of a neuropsychological assessment, Magnetic Resonance Imaging (MRI) of the brain, hearing assessment, tinnitus questionnaires and psychiatric evaluation. Final inclusion decisions were achieved by a multidisciplinary team, including an otolaryngologist, audiologist, neurosurgeon, neurologist, psychiatrist and (neuro-)psychologist.

**Table 1:** Inclusion and exclusion criteria.

Inclusion criteria	Exclusion criteria
*Patients*	*Patient and comorbidities*
Age 18–69 years	Anatomic cause of tinnitus^a^
Willingness to participate	Active otologic diseases
*Tinnitus*	Pregnancy or breast-feeding
Medically refractory	Life expectancy lower than 2 years
Severe (TQ-score ≥47)	*Psychological and cognition*
Chronic (≥2 years) and stable (not intermittent ≥1 year)	DSM-V psychiatric disorders, other than depression or anxiety
Non-pulsatile	Manifestation of depression or anxiety disorder before tinnitus onset
Uni- or bilateral	Active suicidal thoughts or recent attempts
*Hearing*	Cognitive impairment or coping problems
Average pure tone thresholds for 1, 2 and 4 ​kHz ​< ​60 ​dB for each ear	*Contra-indications*
	For DBS due to potential interference^b^
	For MRI
	For surgery

Abbreviations: TQ, Tinnitus Questionnaire; kHz, Kilohertz; dB, Decibel; DSM-V, Diagnostic and Statistical Manual of mental disorders V; DBS, Deep Brain Stimulation; CI, Cochlear Implant; ABI, Auditory Brainstem Implant; MRI, Magnetic Resonance Imaging.

a Examples are vestibular schwannoma, tumour, middle-ear pathology and temporal mandibular disorder.

b Implantable electronic devices with potential interference are CI, ABI, cortical implant.

### Surgery

A two-staged surgical procedure was performed. During the first stage, bilateral DBS leads were implanted in the MGB, and electrodes were externalised for the recording of neurophysiological data. After a few days, the electrodes were internalised and connected to an internal pulse generator (IPG, Activa PC model 37601, Medtronic, Minneapolis, US).

During the first stage, the trajectory of electrode implantation was planned by fusion of a per-operative computed tomography (CT) scan with a pre-operative 3-T (T) MRI scan. The MGB target location was based on the visualisation of the MGB on T_2_-weighted images and coordinates of the MGB in the Schaltenbrand Atlas [16]. A standard stereotactic surgical procedure was conducted under local anaesthesia. Initially, one (three patients) or two (one patient) micro-electrode(s) (InoMed, Emmendingen, Germany) were inserted. Neurophysiological recordings were performed in 0.5–1 ​mm steps from 10 ​mm superior, and maximally 5 ​mm inferior, to the target with a multi-channel system (InoMed, Emmendingen, Germany) [17]. Simultaneously, at each step, a sequence of auditory stimuli was presented with in-ear earbuds. Test electrical stimulation was applied to assess potential direct effects on tinnitus levels and identify any undesired side effects. An experienced DBS neurologist (MJ) evaluated each patient's response during test stimulation.

The final stimulation electrode (model 3389, Medtronic, Minneapolis, US) was placed if an acceptable therapeutic window or absence of side effects was found. In none of the participants modulation of tinnitus was directly observed. In all patients a sensation in the contra-lateral body was experienced if current was increased. All of the subjects were implanted at the originally planned tract. After removal of the stereotactic frame, the fixated electrodes were externalised for neurophysiological recordings, which took place at the neurosurgical medium care unit. A post-operative CT scan was conducted to compare the definite electrode position with the planned trajectory. The second surgery followed after a few days. Under general anaesthesia, the IPG was implanted subcutaneously in the paraumbilical subcutaneous layer and was connected with the internal electrodes.

### Stimulation settings

Stimulation parameters were determined in the optimization phase. During this six-week period, patients visited the outpatient clinic every week to monitor the effect, tolerability, and side effects of the stimulation in daily life. Monopolar stimulation was applied (electrode cathode, IPG anode). The following parameters, with minimum and maximum values, were tested: stimulation frequency (2–200 ​Hz (Hz)), pulse duration (60–450 ​μs (μs)) and stimulation intensity (0–5 ​V (V)). Initially, a monopolar review was conducted for all electrode contacts. These were, from ventral to dorsal, contacts 8, 9, 10 and 11 in the right and contacts 0, 1, 2 and 3 in the left hemisphere. Symmetrical stimulation was started, and the intensity was increased until stimulation-induced side effects occurred. Once the final active contacts were chosen on both sides, stimulation parameters were optimised to achieve the best clinical effect. Optimised DBS settings were determined based on the patent's feedback and clinical observations by MJ. These optimised settings were used during the cross-over phase with stimulation ON. During the unblinded open-label phase, patients were allowed to change the stimulation intensity between individually assigned ranges determined at the outpatient clinic, switch between DBS program settings, or turn the stimulation off.

### Outcome measures

Primary outcome was safety and feasibility of MGB DBS. Safety was assessed by collecting all adverse events, including stimulation-induced side effects. Feasibility was determined based on the ability to recruit a sufficient number of patients and their tolerance of the intervention. Tolerability was assessed through qualitative interviews conducted during each outpatient clinic visit. Secondary outcome measures were tinnitus severity, hearing function, psychological functioning, QoL, and cognitive functioning. Tinnitus severity was assessed using the Tinnitus Functional Index (TFI) [18]. A decrease of 13 or more points in the total score was considered as a clinically significant change [19]. Hearing function was assessed by pure tone and speech audiometry as well as Auditory Brainstem Response (ABR). Psychological functioning was assessed using four questionnaire scores: 1&2 The Hospital Anxiety and Depression Scale (HADS), separately for anxiety and depression, each with a score >10 being considered clinically relevant [20]. 3) The Beck Depression Inventory II (BDI-II) with four grades (0–13 minimal, 14–19 mild, 20–28 moderate, 29–63 severe) [21]. 4) The Beck Anxiety Inventory (BAI) with four grades (0–7 minimal, 8–15 mild, 16–25 moderate, 26–63 severe) [22]. Health-related QoL was measured using the 36-Item Short Form Health Survey (SF-36) [23]. This survey results in a total sum score (0–800) assessing eight domains (0–100 for each): physical functioning, physical role limitations, emotional role limitations, vitality, mental health, social functioning, bodily pain, and general health. A higher score indicates a better QoL. Cognitive functioning was assessed by a neuropsychologist, based on standard tests for naming, verbal memory, mental speed, and executive functioning (flexibility and response inhibition). Adverse events were monitored throughout the study. Data analysis was performed using descriptive statistics as appropriate for this sample size. Z-scores were provided for cognitive data. Reliable change indices (RCI) were calculated for psychological functioning and QoL to determine whether the change in a test score of an individual was significant between two time points (1-year follow-up and baseline) by considering the reliability of the measure (Supplement I).

### Electrode localisation

The location of the implanted DBS electrodes relative to the MGB was reconstructed in 3D (Lead-DBS toolbox v3.0 in MATLAB R2023b) [24]. This reconstruction involved linearly co-registering the post-operative CT scan with the pre-operative T_1_-weighted MRI data [25]. All co-registrations were manually inspected and refined where needed. A brain shift correction step was applied in patients where this was judged to improve alignment [26]. The T_1_-weighted MRI data were then co-registered to the MNI152 NLIN 2009b template using the SyN registration approach [27], transforming the CT scan into the standardised Montreal Neurological Institute (MNI) coordinate space [28]. DBS electrode contacts were automatically reconstructed based on the CT scan using the PaCER algorithm [29], and manually refined if required. The location of the MGB was based on the Jülich Brain Atlas [30], which was already defined in the same MNI coordinate space. A threshold of 0.5 was applied to the MGB atlas, defining the MGB as the regions where at least five out of ten atlas brains showed its presence. This threshold was chosen as a balance between being selective and avoiding excessive restrictiveness. The MNI coordinate system in millimetres (mm) was used to assign x-, y-, and z-coordinates to the centre of the atlas-defined MGB and the electrode contacts of all patients, separately for the left and right side of the brain. Differences in the x-, y- and z-coordinates between the deepest electrode contact and the centre of the atlas-based MGB were calculated to quantify offset in each direction, with the overall separation measured using the Euclidean distance.

## Results

### Study population

Recruitment started in 2021 and this study ended in 2024. Six patients were screened for participation (Fig. 1). Two patients were excluded after screening. One patient was not suited, since his tinnitus was accompanied by a severe form of hyperacusis. Furthermore, his psychiatric comorbidities were not considered stable due to ongoing adjustments of high-dose antidepressants by his psychiatrist. A second patient was excluded after experiencing a positive effect on his tinnitus from wearing a hearing aid during the screening phase. Four patients met the eligibility criteria, underwent surgery, and completed the follow-up. All study participants were male, their level of tinnitus was severe to very severe (grade 3–4) based on the baseline TQ-score. Tinnitus was bilateral and chronic. Patients 1 and 2 had age-related sensorineural hearing loss, while patients 3 and 4 had normal to near-normal hearing with a 4 ​kHz (kHz) notch (Supplement II). Hypertension and psychiatric history were the main comorbidities (Table 2).

**Table 2:** Baseline characteristics.

Characteristics	Patient 1	Patient 2	Patient 3	Patient 4
Age (Y)	54	50	29	56
Sex	Male	Male	Male	Male
PTA AD/AS (dB HL)^a^	28/23	15/15	3/3	18/20
TQ	54 (grade 3)	66 (grade 4)	49 (grade 3)	65 (grade 4)
Tinnitus duration (Y)	9	5.5	7.9	6
Tinnitus lateralisation	Bilateral	Bilateral	Bilateral	Bilateral
Comorbidities	Hypertension, depression	Adjustment disorder	None	Sudden deafness
				AS 9Y ago
Medication	Losartan, Hydrochlorothiazide, Sertraline, Temazepam	Escitalopram	None	Sertraline

Abbreviations: Y, Years; PTA, Pure Tone Average; AD, Auris Dextra; AS, Auris Sinistra; dB, Decibel; HL, Hearing Loss; TQ, Tinnitus Questionnaire; kHz, Kilohertz.

a Patients 1 and 2 had age-related sensorineural hearing loss at baseline, patient 3 had normal hearing with a 4 ​kHz notch of 20 ​dB, and patient 4 had near-normal-hearing with a 4 ​kHz notch of 50 ​dB.

### Safety and feasibility

Recruitment of six participants was without any effort, although recruitment was paused due to the COVID-19 pandemic between the first and second participant. All patients experienced DBS as an acceptable treatment and adhered to the stimulation protocol throughout the trial. No irreversible stimulation-induced side effects occurred. In particular, hearing was not altered, and no novel sounds were induced by the stimulation.

Adverse events were categorised as surgery-related or stimulation-related. All patients encountered surgical-related adverse events. Patients 1 and 3 were preventively treated with oral antibiotics due to wound redness to prevent surgical site infection. Patient 2 underwent surgical revision under general anaesthesia because of cranial wound dehiscence. Patient 4 needed extra surgery to remove guide tubes of the externalised electrodes, which were left behind accidently after internalisation. All surgical-related complications were temporary and had no long-term effects on the health status of the patient. Transient post-operative complaints were headache, nausea and fatigue, which were not present at discharge from the hospital. Patient 3 experienced post-operative change of sensation in his left upper and lower limbs which completely resolved within one month.

Stimulation-induced side effects were observed when stimulation intensity was increased. These side effects included a tingling sensation on the contralateral side of the body, electrical sensation, otalgia, jaw pain, muscle tightness in the shoulder area, dizziness, and headache. These undesired side effects were reversible with decreased stimulation intensity.

### Tinnitus

Secondary outcome measures on tinnitus severity, psychological functioning, and health-related QoL are presented in Table 3. Patients 1 and 4 had clinically relevant improvement of TFI (≥13) at one year follow-up from baseline. Patient 3 improved 7 points on the TFI during this period. Patient 2 had no improvement.

**Table 3:** Tinnitus severity, hearing level, psychological and quality of life scores at baseline, after cross-over phase I, cross-over phase II and 1-year follow-up.

Functioning	Measure	Patient	Baseline	Cross-over (phase I)	Cross-over (phase II)	1-Year follow-up	RCI (1-year - baseline)
Tinnitus severity	**TFI**	1	69	60	49	10	
		2	65	81	76	71	
		3	54	38	38	47	
		4	78	62	58	59	
Hearing level	**PTA AD/AS (dB HL)**	1	28/23	35/33	37/28	30/32	
		2	15/15	18/15	18/13	20/15	
		3	3/3	3/3	5/7	8/8	
		4	18/20	20/18	20/20	22/20	
Psychological	**HADS anxiety**	1	6	6	5	3	−1,26
		2	3	13	10	7	1,69
		3	6	5	5	6	0
		4	11	5	6	7	−1,69
	**HADS depression**	1	9	11	7	0	−3,89^b^
		2	1	10	13	9	3,45^b^
		3	5	6	8	8	1,3
		4	11	7	7	5	−2,59^b^
	**BDI-II**	1	11	16	11	6	−1,41
		2	1	21	17	21	5,66^b^
		3	10	9	9	10	0
		4	12	12	11	7	−1,41
	**BAI**	1	6	7	8	0	−1,57
		2	1	11	7	8	1,83
		3	7	5	7	6	−0,26
		4	9	8	6	8	−0,26
Health-related QoL	**SF-36**^a^	1	692	523	641	752	
		2	735	362	230	317	
		3	622	558	564	618	
		4	512	608	557	554	

Cross-over order for stimulation was OFF-ON for patients 1 and 3 and ON-OFF for patients 2 and 4. Grey shading indicate that stimulation was ON.

Abbreviations*:* TFI, Tinnitus Functional Index; PTA, Pure Tone Average; AD, Auris Dextra; AS, Auris Sinistra; dB, Decibel; HL, Hearing Loss; HADS, Hospital Anxiety and Depression Scale; BDI-II, Beck Depression Inventory II; BAI, Beck Anxiety Inventory; QoL, Quality of Life; SF-36, 36-Item Short Form Health Survey.

a SF-36 score represents a total sum score (0–800) of 8 domains; physical functioning, physical role limitations, emotional role limitations, vitality, mental health, social functioning, bodily pain, and general health.

b Significant reliable change.

### Hearing

Hearing thresholds did not change over time (Table 3, Supplement II). All speech audiometry tests and ABRs were in accordance with tone audiometry results.

### Depression and anxiety

The HADS anxiety and depression scores (Table 3) were only clinically relevant at baseline for patient 4. His scores decreased to a non-clinically relevant level after the cross-over phases and at 1-year follow-up. Patient 2 was the only patient who showed a clinically relevant increase in anxiety and depression scores after surgery. HADS scores of patient 2 were increased during the cross-over phases but normalised to non-clinical relevance at 1-year follow-up. The BDI-II and BAI were categorised minimal to mild at baseline for all patients. BDI-II worsened from minimal to moderate and BAI worsened from minimal to mild for patient 2 after the cross-over phases and at 1-year follow-up. Changes in the absolute scores at 1-year follow-up compared to baseline were clinically relevant for patient 1 and 4, with improved HADS depression scores and for patient 2 with worsened HADS depression and BDI-II scores.

During the final open-label phase, patients 1 and 2 experienced a transient increase in psychological complaints, feeling depressed, frustrated or being anxious. These symptoms did not respond to changes in stimulation settings in either patient. For patient 1, the symptoms were likely related to discontinuation of antidepressant medication (without notifying the study team), since these complaints faded after the psychiatrist reinstated the medication. For patient 2, these symptoms coincided with difficulties in family circumstance.

### Cognition

Cognitive functioning, based on test scores transformed to z-scores presented in Supplement III, was overall assessed as stable or improved over time. Only the Stroop test, a measure for response inhibition, prolonged significantly for patient 1 and card 1 of the Stroop for patient 2 after surgery.

### Quality of life

Health-related QoL, measured with the SF-36 (Table 3) based on eight subdomains (Supplement IV) showed improvement of social functioning for patient 1, reduced physical role limitations for patient 3 and improved physical functioning for patient 4 ​at 1-year follow-up compared to baseline. Bodily pain was worsened with a reliable change for patient 3 after one year. All QoL scores of patient 2 decreased significantly.

### Stimulation parameters

The deepest contact of the left electrode (contact 0) and the right electrode (contact 8) were used for stimulation in all patients during the cross-over and open-label phase. Stimulation parameters of frequency, pulse duration, and stimulation intensity are shown in Table 4 during cross-over with stimulation ON and after 1-year follow-up.

**Table 4:** DBS settings with parameters during cross-over phase and after 1-year follow-up.

	Patient 1		Patient 2		Patient 3		Patient 4	
	Right	Left	Right	Left	Right	Left	Right	Left
**During cross-over**								
Frequency (Hz)	100		100		130		60	
Pulse duration (μs)	60		60		60		60	
Stimulation intensity (V)	1.0	1.0	1.0	1.0	1.4	1.2	1.0	0.6
Contact	8	0	8	0	8	0	8	0
**1-Year follow-up**								
Frequency (Hz)	100		160		180		60	
Pulse duration (μs)	60		60		60		60	
Stimulation intensity (V)	1.6	1.6	1.0	0.9	1.25	1.15	0.3	0.5
Contact	8	0	8	0	8	0	8	0

Deepest electrode contacts were used in all patients for monopolar stimulation with the deepest contact as cathode and the internal pulse generator case as anode.

Abbreviations: DBS, Deep Brain Stimulation; Hz, Hertz; μs, Microseconds; V, Volts.

### Electrode localisation

**Fig. 2:**
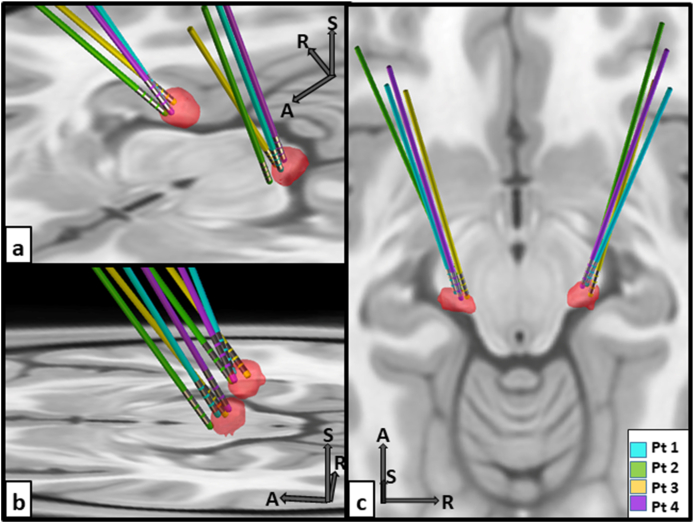
Electrode visualisation. Visualisation of electrode location relative to the atlas-based medial geniculate body (in red) for all patients. Lead-DBS software was used to reconstruct electrode position and the results are displayed on multiple axial views of the MNI152 NLIN 2009b template (a–c). Arrows indicate the superior (S), anterior (A) and right (R) directions for orientation. Abbreviation: Pt, Patient.

A 3D reconstruction of the DBS electrode locations across patients relative to the common atlas-based MGB is presented in Fig. 2; see Supplement V for a video. Electrode and MGB position within the standardised MNI coordinate space are detailed in Table 5. On average, the distance (with standard error of the mean) across patients between the deepest electrode contact and the MGB centre along the x, y-, and z-axes was 0.3 (0.2), 1.6 (0.5) and 0.5 (0.4) mm, respectively. This indicates that the deepest electrode contacts were implanted close to the centre of the atlas-based MGB along the x- (left to right) and z-axis (inferior to superior). However, along the y-axis, the electrodes were consistently positioned anterior to the centre of the atlas-based MGB. This anterior deviation was observed for all electrodes except the right electrode of patient 3 and the left electrode of patient 4 (Table 5). Overall, the greatest Euclidean distance between the deepest electrode contact and the MGB centre was observed in patient 2. This finding was confirmed by the 3D reconstruction, showing the largest distance to the MGB for the electrodes of patient 2 (green electrodes in Fig. 2).

**Table 5:** x-, y- and z-coordinates and Euclidean distance for the active electrode contact and centre of the MGB.

MNI coordinates				Difference (electrode-MGB)			
	x	y	z	x	y	z	Euclidean distance
**MGB**							
Left	−14,5	−25,5	−6,5				
Right	16,5	−25,0	−6,0				
**Patient 1**							
Electrode left	−16,0	−23,9	−5,5	−1,5	1,6	1,0	2,4
Electrode right	17,0	−22,7	−4,7	0,5	2,3	1,3	2,7
**Patient 2**							
Electrode left	−14,8	−22,2	−7,9	−0,3	3,3	−1,4	3,6
Electrode right	16,9	−22,0	−6,3	0,4	3,0	−0,3	3,1
**Patient 3**							
Electrode left	−13,3	−25,0	−6,2	1,2	0,5	0,3	1,3
Electrode right	17,7	−25,1	−5,6	1,2	−0,1	0,4	1,3
**Patient 4**							
Electrode left	−14,9	−25,7	−4,4	−0,4	−0,2	2,1	2,1
Electrode right	15,9	−22,8	−5,7	−0,6	2,2	0,3	2,3
**Average (SEM)**				0,3 (0,2)	1,6 (0,5)	0,5 (0,4)	2,4 (0,3)

MNI coordinate system in millimetres: the origin of the MNI coordinate system is the anterior commissure, x-axis extends from the left side (−) of the brain to the right side (+), y-axis points from posterior (−) to anterior (+), z-axis points from inferior (−) to superior (+).

The left column shows the MNI coordinates of the centre of the left and right atlas-based MGB and of the deepest (active) contact of the left and right electrodes per patient.

The right column shows the difference between the deepest (active) electrode contact and centre of the MGB, separated by hemisphere and patient. The Euclidean distance combines the offset between electrode and MGB along the x-, y- and z-axes.

Abbreviations: MGB, Medial Geniculate Body; MNI, Montreal Neurological Institute; SEM, Standard Error of the Mean.

Supplementary video related to this article can be found at https://doi.org/10.1016/j.neurot.2025.e00783

The following is the supplementary data related to this article:

Multimedia component 2 Multimedia component 2

## Discussion

In the present pilot-study neuro-surgical intervention and stimulation were well tolerated in all patients and did not result in altered hearing or induction of sounds. In contrast, DBS of a locus of caudate neurons triggers new sounds [31]. Although surgery-related adverse events occurred in all patients, all were resolved within a month without lasting effects. In this case series, all patients experienced transient, stimulation amplitude-dependent paraesthesia, which disappeared immediately upon lowering the stimulation amplitude. The occurrence of paraesthesia is a common transient side effect also seen in stimulation of the ventral intermediate nucleus (VIM) and subthalamic nucleus (STN) for movement disorders [32]. The paresthesias may be explained by two potential mechanisms. First, the electrical field generated during stimulation could spread to the medial lemniscus, a white matter tract adjacent to the MGB that transmits tactile sensory information. Second, animal studies have demonstrated that approximately 25 ​% of neurons in the medial subdivision of the MGB respond to tactile stimuli [33]. This multisensory property of the MGB may contribute to the tactile sensations reported during MGB stimulation. Recruitment of suitable and sufficient participants for the study was feasible. After one year, tinnitus distress (as measured with TFI) was reduced by MGB DBS for patient 1 and 4. Patient 3 showed some improvement, though this improvement did not reach the minimal clinically relevant difference on the TFI. In contrast, tinnitus in patient 2 deteriorated. In summary, the main outcome of this study was that bilateral MGB DBS appears to be a safe and feasible potential therapy for refractory tinnitus.

Changes in tinnitus distress (TFI scores) over time varied across patients. Patients 1 and 4 experienced improvements during stimulation ON in the cross-over phase. Patient 1 started with stimulation OFF and showed responses only during stimulation ON. Patient 4 started the first cross-over phase with stimulation ON. The positive effect on tinnitus distress remained in the second cross-over phase of stimulation OFF. Patient 3, however, fluctuated in his response. He already showed a clinically significant improvement of the TFI score at the first cross-over phase with stimulation OFF. This improvement remained consistent at the second cross-over phase with stimulation ON but was not clinically significant at the one-year follow-up. These observations may indicate the influence of several factors. First, a microlesion effect of the surgery could have resulted in improved tinnitus outcome for patient 3 during the first cross-over phase. The effect of DBS on tinnitus may not be immediate, but may require longer term stimulation to induce plastic changes, as also known in other disorders [34]. Secondly, it was shown that patients can immediately detect when stimulation is turned on or off in several targeted brain areas [35]. Lastly, a placebo effect or a reduction of therapeutic benefit (nocebo), due to the uncertainty of being ON or OFF during the cross-over phases, may have played a role [36]. These factors are important to consider in the design of a study that will assess the clinical effect of MGB DBS on tinnitus.

Patient 2 was a non-responder. He experienced psychological symptoms early in the study after the first cross-over phase, due to family distress. His increased depressive symptoms remained until the end of the study, and were not influenced by stimulation ON or OFF. This indicates that in the most complex syndromic presentation of tinnitus, including psychiatric comorbidities, there is an unmet clinical need for integrated care.

In all patients, the deepest (active) contact of both DBS leads was positioned close to the centre of the atlas-based MGB. The deviations in placement we observed can likely be attributed to discrepancies between atlas coordinates and the individually estimated MGB locations, as the precise location of the human MGB varies across individuals. This variation is visible in the T_2_ data and is considered during surgical planning. Beyond these discrepancies, we consistently observed an anterior deviation in the electrode position relative to the MGB atlas coordinates. An important contributor to this anterior deviation from the intended target is the orientation of the basal vein of Rosenthal, which passes through the ambient cistern immediately posterior to the MGB. To avoid this, the DBS target was placed as anteriorly as possible, likely accounting for the observed relative shift in electrode position. Despite these considerations, it is noteworthy that the distance from the active DBS contacts to the centre of the MGB was considerably larger for patient 2 than the others. This distance could also be a contributing factor to the lack of responses in patient 2.

The study findings should be interpreted cautiously due to several limitations. Firstly, the small sample size, which was a self-evident limitation of the nature of a feasibility study. The small patient number restricted the generalizability of the findings and limited statistical power for detecting clinically meaningful effects. Secondly, clinical outcome on tinnitus could have been influenced by many factors. The possibility of lesion effects associated with stereotactic electrode implantation could not be entirely excluded, as surgical intervention itself might have contributed to observed changes in tinnitus symptoms. However, tinnitus relief was not observed during the first weeks of recovery after surgery with stimulation OFF. Moreover, the study design allowed for the possibility of placebo responses or expectation effects, making it difficult to disentangle stimulation-related improvements from non-specific influences. Patient blinding for stimulation was not possible due to directly experienced sensations of stimulation. Furthermore, tinnitus reporting is inherently subjective, and the outcome measures relied predominantly on self-reported scales, increasing the risk of bias due to psychological or situational factors. Another limitation was the duration of the optimization phase, which was limited to six weeks with six visits. These restricted visits may not have allowed sufficient time to fully explore the broad parameter space of amplitude, pulse width, frequency, and cathode/anode configuration, as highlighted by prior studies, in which optimization could take five months to over a year with ongoing adjustments and patient-led programming [37]. Outcome could have potentially been better if the optimization phase would have been longer. Finally, the optimal DBS target within the MGB remains uncertain, with anatomical variability and distance from the intended target potentially affecting individual outcomes. Different steps in stereotactic surgery, fusion of images and electrode reconstruction could lead to an unintended target error (TE). For frame-based DBS, the TE has decreased since the beginning of this century, from an average of 4 ​mm to between 1 and 2 ​mm [38]. Nevertheless, a small TE of 1–2 ​mm is of importance when implanting a DBS electrode into a small structure like the MGB (4 x 5 ​× ​4.5 ​mm^3^) [39].

The MGB is characterised by three main subdivisions: the dorsal (MGD), medial (MGM), and ventral (MGV) divisions [40]. The MGV is part of the lemniscal auditory pathway, and primarily projects to the primary auditory cortex (PAC) [41]. In contrast, the MGD and MGM integrates auditory input with non-auditory input [42]. Unlike the MGV, both subdivisions project to the non-PAC and the amygdala [43]. The amygdala is associated with emotional processing of tinnitus [44], and has connections to the nucleus accumbens (NAc) and hippocampus, both of which have been implicated in tinnitus [45]. Probably, the active DBS contacts were positioned closest to the anterior portion of MGD [12]. In future studies, the optimal position of the active DBS contact within the MGB needs to be further elucidated. In the current study, DBS leads with ring contacts were used. However, newer DBS leads have contacts that are divided into segments, allowing steering of the stimulation current towards specific directions. This potentially minimizes side effects and will allow more precise modulation of electric fields within the MGB.

In conclusion, this study showed that bilateral MGB DBS is safe and feasible for patients with treatment refractory tinnitus, with promising potential clinical benefits. Both the surgical and stimulation intervention were well tolerated. A follow-up study is needed to further evaluate the effectiveness of MGB DBS on refractory tinnitus.

## Author Contributions

**Shabnam Babakry**: Contributed data or analysis tools; Performed the analysis; Wrote the paper.

**Jana V.P. Devos**: Conceived and designed the analysis; Collected the data; Contributed data or analysis tools; Wrote the paper.

**Catharine A. Hellingman**: Conceived and designed the analysis; Contributed data or analysis tools; Wrote the paper.

**Linda Ackermans**: Wrote the paper.

**Jasper V. Smit**: Conceived and designed the analysis; Wrote the paper.

**Michelle Moerel**: Contributed data or analysis tools; Performed the analysis; Wrote the paper.

**Carsten Leue**: Conceived and designed the analysis; Contributed data or analysis tools; Wrote the paper.

**Annelien A. Duits**: Contributed data or analysis tools; Wrote the paper.

**Yasin Temel**: Conceived and designed the analysis; Wrote the paper.

**Marcus L.F. Janssen**: Conceived and designed the analysis; Contributed data or analysis tools; Wrote the paper.

## Declaration of competing interest

The authors declare that they have no known competing financial interests or personal relationships that could have appeared to influence the work reported in this paper.
