# Supplementary material for: Deep brain stimulation of the medial geniculate body for refractory tinnitus: A feasibility study
Source: Neurotherapeutics. 2025 Nov 18;23(1):e00783. doi: 10.1016/j.neurot.2025.e00783 (PMC12976501; doi:10.1016/j.neurot.2025.e00783)
Supplement: Multimedia component 1 [file mmc1.pdf]

Supplemental material

Supplement I. Reliable change index

RCI was calculated for psychological functioning and quality of life with the following formulas of Jacobson and Truax.(1) $RCI = \frac{x_{post} - x_{pre}}{\sqrt{2S_E^2}}$ $S_E = SD\sqrt{1 - r_{xx}}$

Reliable change was considered in case of a RCI > 1.96 or a RCI < -1.96. The calculation was based on standard deviations of HADS,(2) BDI-II,(3) BAI,(4) and SF-36 (5) in tinnitus populations and on Cronbach's alpha reliability of HADS,(6) BDI-II, (6) BAI,(7) and SF-36.(8)

Abbreviations: RCI, Reliable Change Index; HADS, Hospital Anxiety and Depression Scale; BDI-II, Beck Depression Inventory II; BAI, Beck Anxiety Inventory; SF-36, 36-Item Short Form Health Survey.

Supplement II. Tone and speech audiograms

Tone and speech audiograms at baseline, after surgery, cross-over phase I, cross-over phase II and 1-year follow-up. Audiometry provided hearing thresholds. All speech audiometry tests and auditory brainstem responses were in accordance with tone audiometry results.

A. Patient 1, B. Patient 2, C. Patient 3, D. Patient 4

A

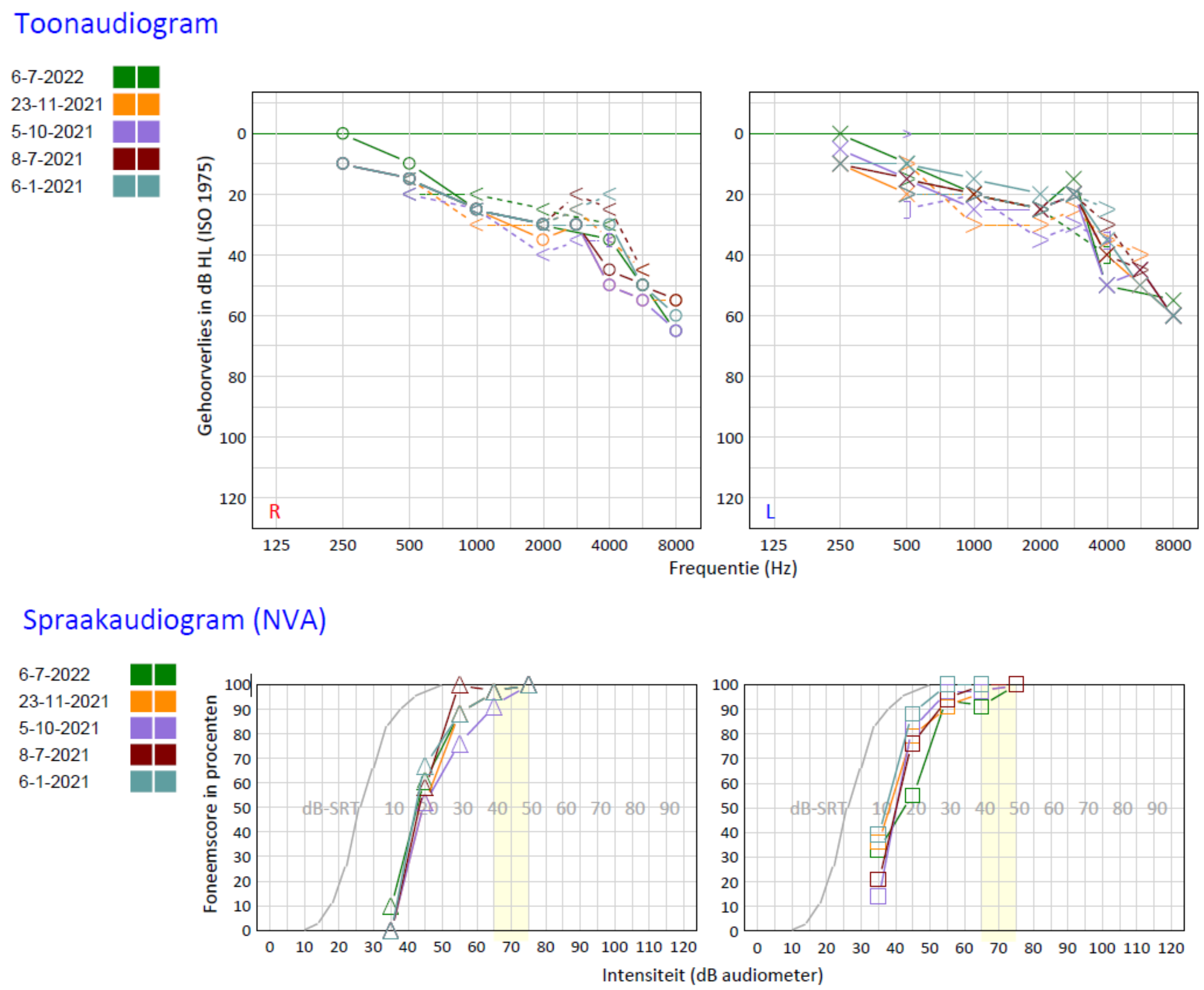

B

Toonaudiogram

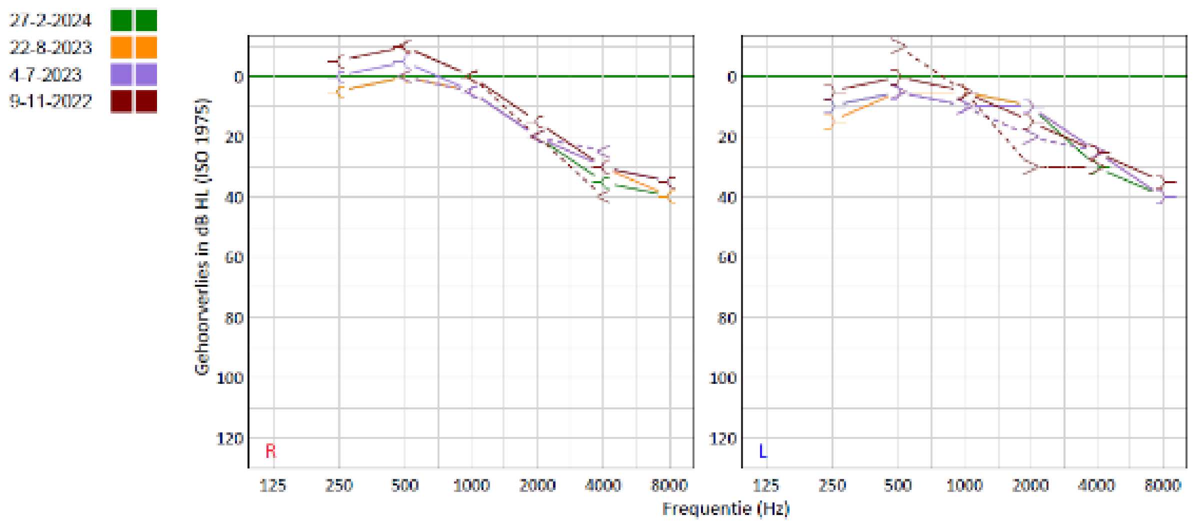

Spraakaudiogram (NVA)

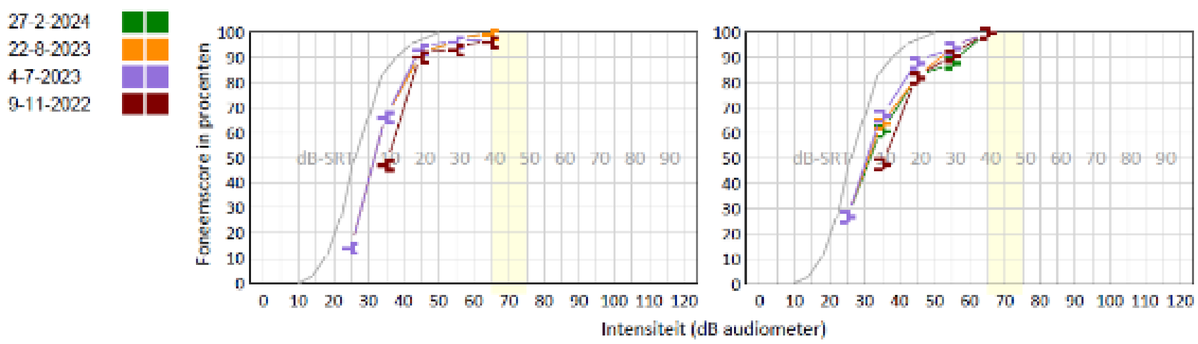

C

Toonaudiogram

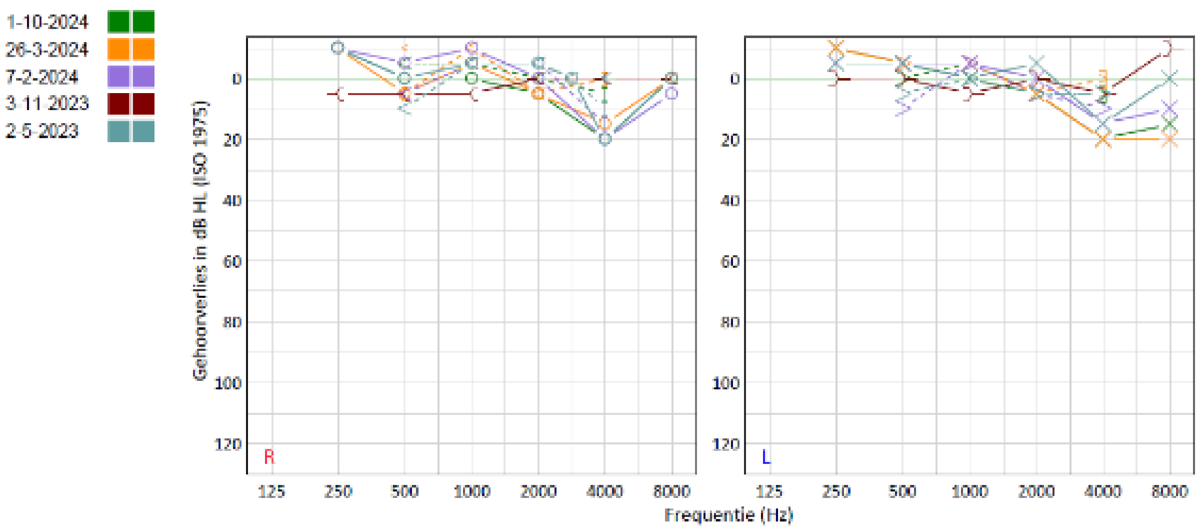

Spraakaudiogram (NVA)

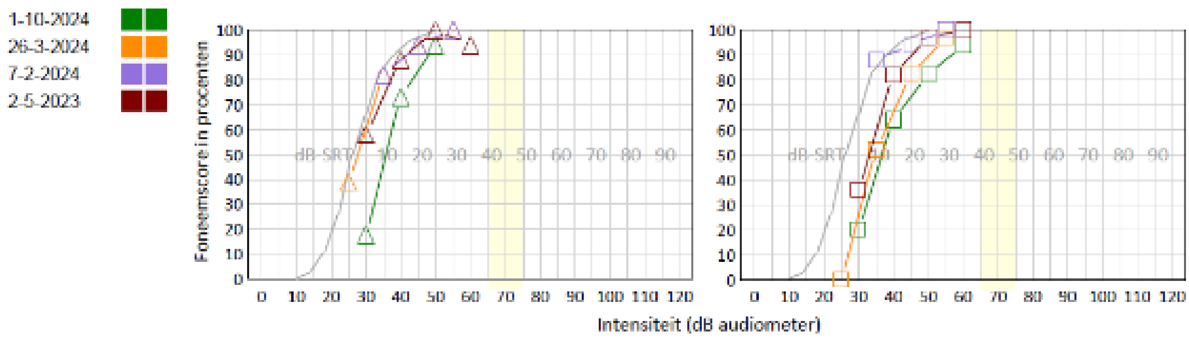

D

Toonaudiogram

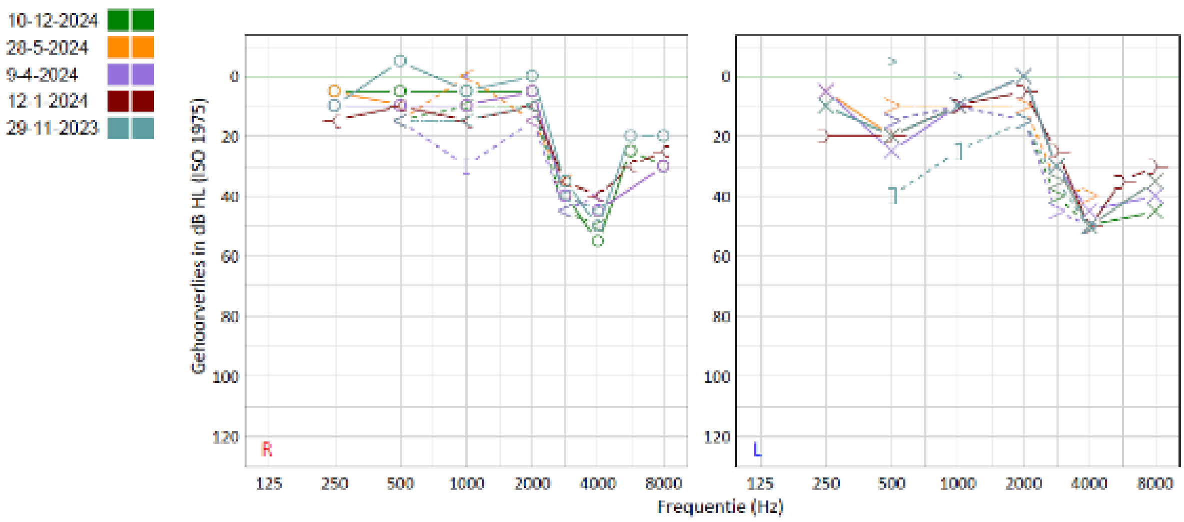

Spraakaudiogram (NVA)

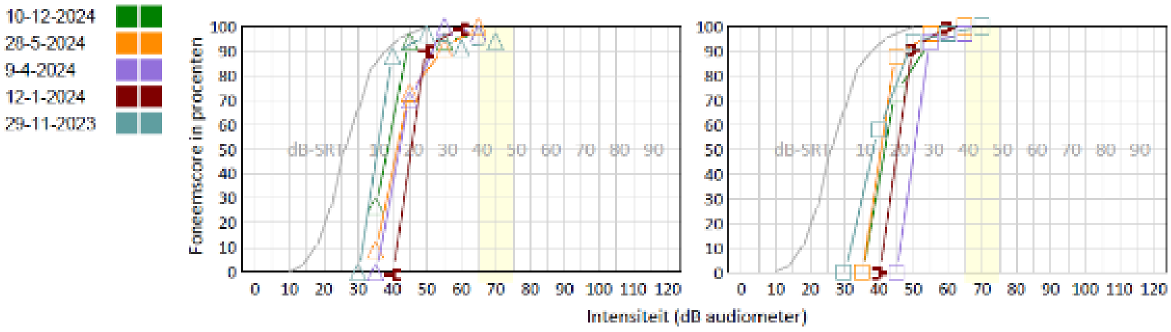

Supplement III. Cognitive functioning

Measure	Patient	Baseline (z-score)	Cross-over phase I (z-score)	Cross-over phase II (z-score)	1-Year follow-up (z-score)
**Boston Naming Test**					
	1	28,5 (0,5)	28,5 (0,5)	29 (0,5)	n.a.
	2	28 (0,4)	28 (0,4)	30 (0,85)	27 (0,2)
	3	29 (0,63)	29 (0,63)	29 (0,63)	30 (0,85)
	4	28 (0,4)	27 (0,2)	28 (0,4)	26 (0)
**Verbal and letter fluency**					
Semantic: animal/job	1	23/18 (-0,51/-0,57)	21/19 (-0,8/0)	29/17 (0,8/-0,5)	n.a.
	2	26/17 (0,2/-0,5)	24/22 (-0,12/0,56)	20/19 (-0,79/-0,05)	20/21 (-0,77/0,38)
	3	27/20 (0,4/0,5)	31/17 (1,1/-0,2)	28/21 (0,6/0,7)	28/19 (0,6/0,2)
	4	25/14 (0,4/-0,7)	20/15 (-0,6/-0,5)	20/15 (-0,6/-0,5)	20/13 (-0,6/-1,0)
Phonological	1	39 (-0,2)	43 (0,2)	41 (0)	n.a.
	2	44 (0,7)	43 (0,6)	40 (0,3)	42 (0,5)
	3	34 (-0,3)	37 (0)	38 (0,1)	39 (0,2)
	4	20 (-1,7)	18 (-1,9)	21 (-1,6)	33 (-0,4)
**15-Word Test**					
Total correct 5 rounds	1	22 (-3,18)	45 (-0,4)	41 (-0,9)	n.a.
	2	52 (0,8)	48 (0,30)	52 (0,8)	54 (1)
	3	58 (1,5)	52 (0,8)	57 (1,4)	60 (1,8)
	4	27 (-2,2)	36 (-1,1)	33 (-1,4)	34 (-1,3)
Delayed recall	1	10 (0,0)	11 (0,4)	10 (0)	n.a.
	2	11 (0,6)	11 (0,6)	12 (1)	12 (1)
	3	14 (1,8)	12 (1,0)	13 (1,4)	15 (2,2)
	4	4 (-2)	7 (-0,8)	6 (-1,2)	7 (-0,8)

**Trail Making Test (s)**					
Trail A	1	47 (-1,8)	78 (-3,0)	60 (-2,6)	n.a.
	2	24 (0,7)	23 (0,8)	24 (0,8)	23 (0,9)
	3	18 (1,3)	20 (1,4)	18 (1,8)	18 (1,8)
	4	44 (-1,1)	32 (-0,2)	32 (-2,0)	37 (-0,65)
Trail B	1	107 (-1,5)	113 (-1,7)	121 (-1,9)	n.a.
	2	97 (-1,05)	67 (0,0)	38 (1,8)	40 (1,7)
	3	41 (1,0)	41 (1,7)	28 (2,75)	40 (1,7)
	4	87 (-0,5)	60 (0,5)	73 (-0,1)	87 (-0,65)
**Stroop Color-Word Test (s)**					
Subtest 1	1	50 (-1,47)	68 (-2,9)	61 (-2,5)	n.a.
	2	41 (-0,2)	59 (-2,2)	52 (-1,6)	58 (-2,2)
	3	54 (-1,9)	48 (-1,3)	45 (-0,9)	52 (-1,7)
	4	53 (-1,6)	56 (-1,9)	54 (-1,7)	64 (-2,5)
Subtest 2	1	69 (-1,84)	75 (-2,0)	69 (-1,6)	n.a.
	2	54 (-0,1)	61 (-0,8)	57 (-0,4)	62 (-0,9)
	3	52 (0)	50 (0,3)	48 (0,6)	60 (-0,9)
	4	79 (-2)	75 (-1,8)	79 (-2,0)	86 (-2,4)
Subtest 3	1	110 (-1,44)	114 (-1,3)	114 (-1,3)	n.a.
	2	74 (0,8)	76 (0,7)	72 (1,0)	75 (0,8)
	3	67 (1,1)	70 (0,8)	62 (1,0)	73 (0,6)
	4	112 (-0,9)	117 (-1,1)	123 (-1,3)	112 (-0,9)

Different tests with z-scores for cognitive functioning at baseline, after cross-over phase I, cross-over phase II and 1-year follow-up. Cross-over order for stimulation was OFF-ON for patient 1 and 3 and ON-OFF for patient 2 and 4. The values in grey indicate that stimulation was ON.

Cognitive functioning was assessed by the psychologist based on different kind of tests: Boston naming test;(9) verbal and letter fluency, semantic (animal and job categories) as well as phonological (sum of scores of the letters D, A and T), for procedure description and reference norms, see Capitani et al.(10);

15-word test with a score for the learning curve, including a total score of five rounds, and with a score for delayed recall;(11) trail making test in seconds with standard trail A and B(12) and the Stroop color-word test in seconds consisting of three standard subtests.(13)

Abbreviation: s, Seconds.

Supplement IV. Health-related quality of life

SF-36 domain	Patient	Baseline	Cross-over (phase I)	Cross-over (phase II)	1-Year follow-up	RCI (1 year - baseline)
Physical functioning	1	100	100	100	100	0,00
	2	100	65	5	65	-5,06*
	3	100	95	100	100	0,00
	4	85	90	90	100	2,17*
Physical role limitations	1	100	50	75	100	0,00
	2	100	25	0	25	-3*
	3	50	25	25	100	2*
	4	25	50	0	0	-1,00
Emotional role limitations	1	100	67	100	100	0,00
	2	100	67	67	33	-3,07*
	3	100	100	100	100	0,00
	4	100	100	100	100	0,00
Vitality	1	60	50	65	80	1,72
	2	80	20	40	35	-3,87*
	3	55	60	50	45	-0,86
	4	55	50	60	65	0,86
Mental health	1	84	56	68	92	0,64
	2	80	32	48	44	-2,89*
	3	72	60	64	60	-0,96
	4	56	80	76	76	1,61
Social functioning	1	63	50	88	100	3,76*
	2	100	25	0	25	-7,62*
	3	75	63	75	75	0,00
	4	63	75	88	75	1,22

Bodily pain	1	90	65	80	100	1,42
	2	90	78	20	55	-4,99*
	3	100	100	90	78	-3,13*
	4	68	78	78	68	0,00
General health	1	95	85	65	80	1,94
	2	85	50	50	35	-9,7*
	3	70	55	60	60	-1,94
	4	60	85	65	70	1,94
**Total sum score**	1	692	523	641	752	
	2	735	362	230	317	
	3	622	558	564	618	
	4	512	608	557	554	

8 domains scores of the SF-36 at baseline, after cross-over phase I, cross-over phase II and 1-year follow-up. Each domain of the SF-36 score has a score between 0 and 100%, a higher percentage indicating a better quality of life. Cross-over order for stimulation was OFF-ON for patient 1 and 3 and ON-OFF for patient 2 and 4. The values in grey indicate that stimulation was ON. RCI was calculated for the significance of change in the 1-year follow-up score and baseline score.

* = significant reliable change.

Abbreviation: SF-36, 36-Item Short Form Health Survey; RCI, Reliable Change Index.

Supplement V

3D Movie of the electrodes in relation to the medial geniculate body.

References

1. Jacobson NS, Truax P. Clinical significance: a statistical approach to defining meaningful change in psychotherapy research. J Consult Clin Psychol. 1991;59(1):12-9.
2. Adoga AA, Kokong DD, Nimkur TL, Okwori ET. The impact of tinnitus on adult Nigerians: health related Quality of Life assessment of sufferers using the Hospital Anxiety and Depression Scale (HADS) and the RAND-36 item health survey 1.0 questionnaire. Int Tinnitus J. 2015;19(2):26-32.
3. Ooms E, Meganck R, Vanheule S, Vinck B, Watelet JB, Dhooge I. Tinnitus severity and the relation to depressive symptoms: a critical study. Otolaryngol Head Neck Surg. 2011;145(2):276-81.
4. Karaaslan O, Kantekin Y, Hacimusalar Y, Dagistan H. Anxiety sensitivities, anxiety and depression levels, and personality traits of patients with chronic subjective tinnitus: a case-control study. Int J Psychiatry Clin Pract. 2020;24(3):264-9.
5. Haider HF, Ribeiro SF, Hoare DJ, Fialho G, Hall DA, Antunes M, et al. Quality of Life and Psychological Distress in Portuguese Older Individuals with Tinnitus. Brain Sci. 2021;11(7).
6. Fuchten D, Smit AL, Stegeman I. Examining the overlap between tinnitus and depression questionnaires-protocol for an ICF based content analysis. Front Neurol. 2024;15:1376826.
7. Vazquez Morejon AJ, Vazquez-Morejon Jimenez R, Zanin GB. Beck Anxiety Inventory: psychometric characteristics in a sample from the clinical Spanish population. Span J Psychol. 2014;17:E76.
8. Scott KM, Tobias MI, Sarfati D, Haslett SJ. SF-36 health survey reliability, validity and norms for New Zealand. Aust N Z J Public Health. 1999;23(4):401-6.
9. Kaplan EF, Goodglass H, Weintraub S. The Boston Naming Test. second ed. Philadelphia: Lea & Febiger; 1983.
10. Capitani E, Rosci C, Saetti MC, Laiacona M. Mirror asymmetry of Category and Letter fluency in traumatic brain injury and Alzheimer's patients. Neuropsychologia. 2009;47(2):423-9.
11. Saan RJ, Deelman BG. De nieuwe 15-woorden test A en B. Neuropsychologische Diagnostiek. Lisse, The Netherlands: Swets and Zeitlinger; 1998.
12. Tombaugh TN. Trail Making Test A and B: normative data stratified by age and education. Arch Clin Neuropsychol. 2004;19(2):203-14.
13. Stroop JR. Studies of interference in serial verbal reactions. J Exp Psychol. 1935;18:643-62.
